# Sinusoidal Displacement Describes Disorder in CsPbBr_3_ Nanocrystal Superlattices

**DOI:** 10.1021/acsnano.5c20745

**Published:** 2026-01-17

**Authors:** Umberto Filippi, Stefano Toso, Matheus Gomes Ferreira, Lorenzo Tallarini, Yurii P. Ivanov, Francesco Scattarella, Simone Lauciello, Vahid Haghighat, Huaiyu Chen, Megan Landberg, Giorgio Divitini, Jesper Wallentin, Cinzia Giannini, Liberato Manna, Dmitry Baranov

**Affiliations:** † 121451 Istituto Italiano di Tecnologia, Via Morego 30, 16163 Genova, Italy; ‡ International Doctoral Program in Science, Università Cattolica del Sacro Cuore, Brescia 25121, Italy; § Division of Chemical Physics and NanoLund, Department of Chemistry, 5193 Lund University, P.O. Box 124, SE-221 00 Lund, Sweden; ∥ Department of Chemical Engineering, Massachusetts Institute of Technology, Cambridge, Massachusetts 02139, United States; ⊥ Istituto di Cristallografia (CNR-IC), via Amendola 122/O, Bari 71026, Italy; # MAX IV Laboratory, 5193 Lund University, 22100 Lund, Sweden; 7 Synchrotron Radiation Research and NanoLund, Department of Physics, 5193 Lund University, 22100 Lund, Sweden

**Keywords:** Lead Halide Perovskite, Nanocrystal, Colloidal Softness, Superlattice, Disorder, GIWAXS, GISAXS

## Abstract

Disorder is an intrinsic feature of all solids, from crystals of atoms to superlattices of colloidal nanoparticles. Unlike atomic crystals, in nanocrystal superlattices, a single misplaced particle can affect the positions of neighbors over long distances, leading to cumulative disorder. This elusive form of collective particle displacement leaves clear signatures in diffraction, but little is known about how it accumulates and propagates throughout the superlattice. Here we rationalize the propagation and accumulation of disorder in a series of CsPbBr_3_ nanocrystal superlattices by using synchrotron grazing incidence small- and wide-angle X-ray scattering. CsPbBr_3_ nanocrystals of colloidal softness *S* in the range of 0.3–0.7 were obtained by preparing particles with different sizes and ligand mixtures consisting of oleic acid and primary amines of variable lengths. Most diffraction patterns showed clear signatures of anisotropic disorder, with multilayer diffraction characteristics of high structural coherence visible only for the {100} axial directions and lost in all other directions. As the softness decreased, the superlattices transitioned to a more ordered regime where small-angle diffraction peaks became resolution-limited, and superlattice multilayer diffraction appeared for the (110) diagonal reflections. To rationalize these anisotropies in structural coherence and their dependence on superlattice softness, we propose a sinusoidal displacement model where longitudinal and transverse displacements modulate nanocrystal positions. The model explains experimental observations and advances the understanding of disorder in mesocrystalline systems as they approach the limits of structural perfection.

## Graphical abstract

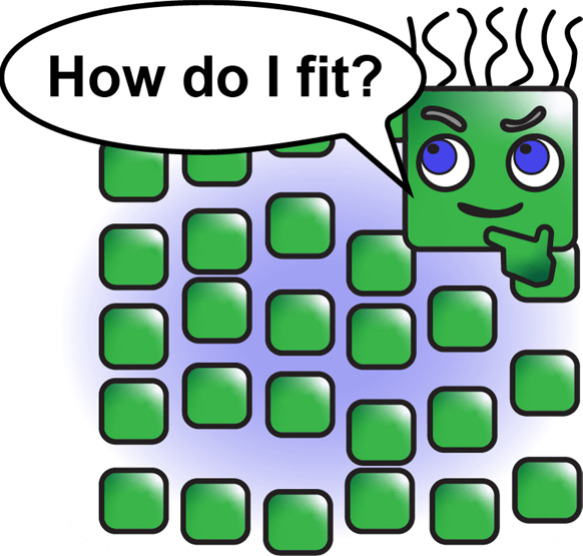

Colloidal nanocrystals are a compelling case of complex building blocks that can spontaneously self-organize into highly ordered three-dimensional solids (nanocrystal-based superlattices), and they attract intense interest because of emergent properties and applications in optoelectronics.^1–4^ Nanocrystal-based superlattices exhibit disorder, owing to nanocrystal size and shape polydispersity, variations in ligand coverage, and weak interparticle forces.^5^ In diffraction experiments, this disorder manifests as peak broadening in higher order reflections. This is a signature of cumulative disorder, where displacement propagates through the structure as each particle position is influenced by that of the neighbors.^6,7^ This is the main reason why nanocrystal-based superlattices are studied by small-angle X-ray scattering, as superlattice interference is lost at wide angles.

However, colloidal perovskite nanocrystals with well-defined cubic shapes have been shown to assemble into superlattices with exceptional structural coherence, manifested by sharp and complex satellite peaks at wide angles.^8–14^ These features suggest an intricate picture of disorder in which precise superlattice periodicity and cumulative displacement can coexist. This raises the question of how positional correlations between neighboring nanocrystals propagate in different spatial directions within the superstructure and, more generally, stimulates a discussion about the nature of disorder in nanoparticle assemblies. To address these questions, one needs to measure both small- and wide-angle X-ray scattering in multiple lattice directions on a series of high-quality superlattice samples with varying disorder. To this end, we performed experiments on perovskite nanocrystal superlattices at the ForMAX beamline of MAX IV, which allows simultaneous collection of grazing-incidence small- and wide-angle X-ray scattering (GISAXS/GIWAXS) patterns with high intensity and angular resolution. This setup provides complementary information, capturing both the average nanometric structure of superlattices (GISAXS) and the interference effects that reveal local structural coherence and disorder (GIWAXS).

Another piece of the puzzle comes from controlling the amount of disorder. Recently, some of us have reported that adopting mixed-ligand passivation enables control of disorder in CsPbBr_3_ superlattices.^14^ The mixed ligand passivation consists of oleic acid with aliphatic amines of variable length, where shorter amines lead to enhanced structural coherence in superlattices. Here, we build on this approach to tune the colloidal softness *S* of nanocrystals, defined as the ratio between the ligand shell thickness and the edge length of nanocrystals (*S* = *L*/NC_edge_).^15–18^ The studied softness range spans from *S* = 0.3 (9.5 nm particles with oleic acid and octylamine) to *S* = 0.7 (5.8 nm particles with oleic acid and oleylamine), and disorder increases with increasing *S*, as evidenced by broadening of the GISAXS spots (Scheme 1).

**Scheme 1:**
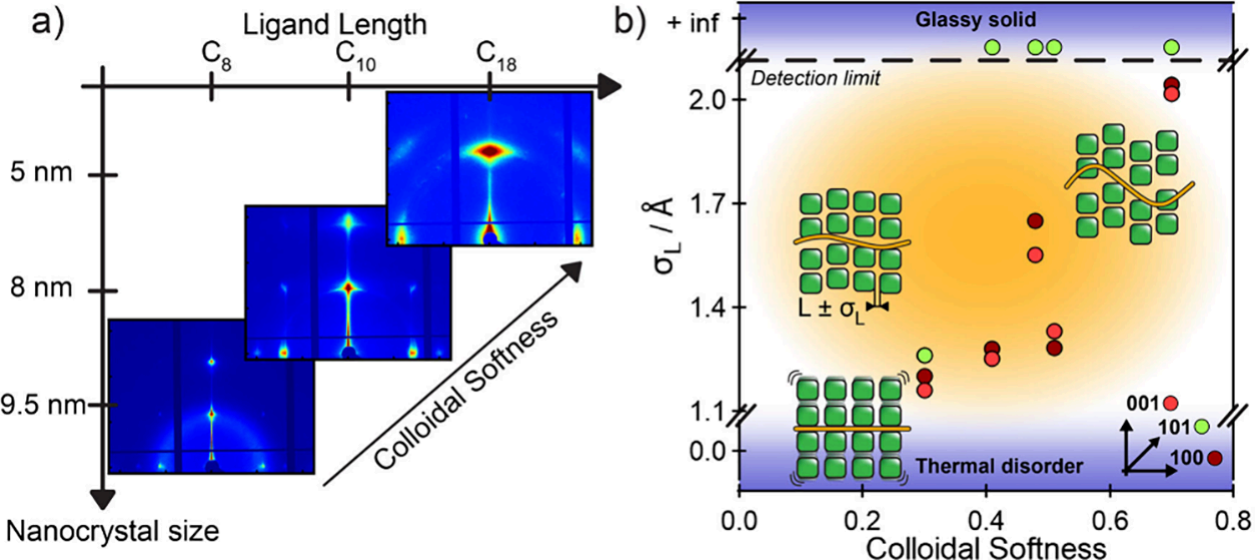
(a) Synchrotron GISAXS Patterns of Three Representative CsPbBr_3_ Nanocrystal Superlattices as a Function of Ligand Length and Nanocrystal Size, Connected through Colloidal Softness (*S*), (b) Regimes of the Structural Disorder of Nanocrystal Superlattices as a Function of the Average Nanocrystal Longitudinal Displacement (σ_L_) and *S* ^a^

a In the illustration, σ_L_ = 0 corresponds to the limit where nanocrystal positions are affected only by thermal-like (uncorrelated) fluctuations, and the σ_L_ → +∞ limit, a “superlattice catastrophe,” corresponds to the case of a short-range order between nanocrystals, as in glassy solids.^19^ The sinusoidal displacement model (wavy orange lines) captures the different disorder regimes through the amplitude, wavelength, and direction (transversal or longitudinal) of the oscillations of nanocrystal positions.

The experiments reported below provide evidence of high structural coherence for both in- and out-of-plane GIWAXS signals along the {100} directions of the CsPbBr_3_ superlattices. Except for the sample with the lowest *S* value (*S* = 0.3), the collective X-ray interference is absent for other diffraction spots in the rest of the samples. In the *S* = 0.3 sample, the signatures of satellite peaks were observed for the GIWAXS (101) reflection. These observations raise the paradox of superlattices that are highly ordered along the main lattice directions, and yet appear disordered along the directions given by their linear combinations. Such observations prompted us to develop a sinusoidal displacement model consisting of longitudinal and transversal components. The longitudinal component affects the face-to-face interparticle distance along the main lattice directions, and the transversal component affects the lateral displacement of nanocrystal rows and columns. The model explains the experimental evidence of anisotropic structural coherence while preserving the intrinsically cumulative nature of disorder in nanocrystal superlattices. This sinusoidal description of disorder in nanocrystal solids can likely be extended to other materials. The results deepen insight into the structure of nanocrystal assemblies and rationalize colloidal softness as a powerful tool for superlattice engineering.

## Results and Discussion

### Nanocrystals with Mixed Ligands

The study was done on a series of superlattices grown from CsPbBr_3_ nanocrystals capped with mixed ligands consisting of oleic acid and amines of different lengths: oleylamine (C_18_), dodecylamine (C_12_), decylamine (C_10_), and octylamine (C_8_).^14^ The starting nanocrystals were synthesized using a common hot-injection method, reacting cesium oleate with PbBr_2_ solubilized in a hot mixture of ligands and 1-octadecene, as summarized in the Methods. Representative high-resolution scanning transmission electron microscopy high-angle annular dark field (STEM-HAADF) images of the nanocrystals are shown in Figures 1 and S1 in Supporting Information (SI). A shortening of the interparticle distance is noticeable, as expected from the incorporation of progressively shorter amines into the ligand shell of the nanocrystals. Upon drying concentrated dispersions on silicon or similar substrates (see Methods), the nanocrystals produce three-dimensional superlattices, with representative scanning electron microscopy (SEM) images of the tilted top view (8 nm-C_18_ nanocrystals) shown in Figure 1e and of a high-resolution region in Figure 1f (see also Figure S2 for HRSEM comparison of all superlattices). The edge lengths of the nanocrystals are approximately 8 nm for C_18_, C_12_, and C_10_ ligands, and 9.5 nm for C_8_ ligands, placing them in the intermediate quantum confinement regime. For comparison, a batch of quantum-confined nanocrystals with an edge length of 5.8 nm (sample 5 nm-C_18_) was synthesized by adapting the protocol reported by Dong et al.^20^ with modifications.^21^ Figure 1g shows the absorption spectra of dilute nanocrystal dispersions, illustrating the intermediate and strong confinement regimes. The colloidal softness was calculated for all samples using the nanocrystal edge length determined from TEM. The *S* values corresponding to the series of studied samples are 0.70 (5 nm-C_18_), 0.48 (C_18_), 0.51 (C_12_), 0.41 (C_10_), and 0.3 (C_8_). Similar values of softness were determined independently from multilayer diffraction analyses (Table 1).

**Figure 1:**
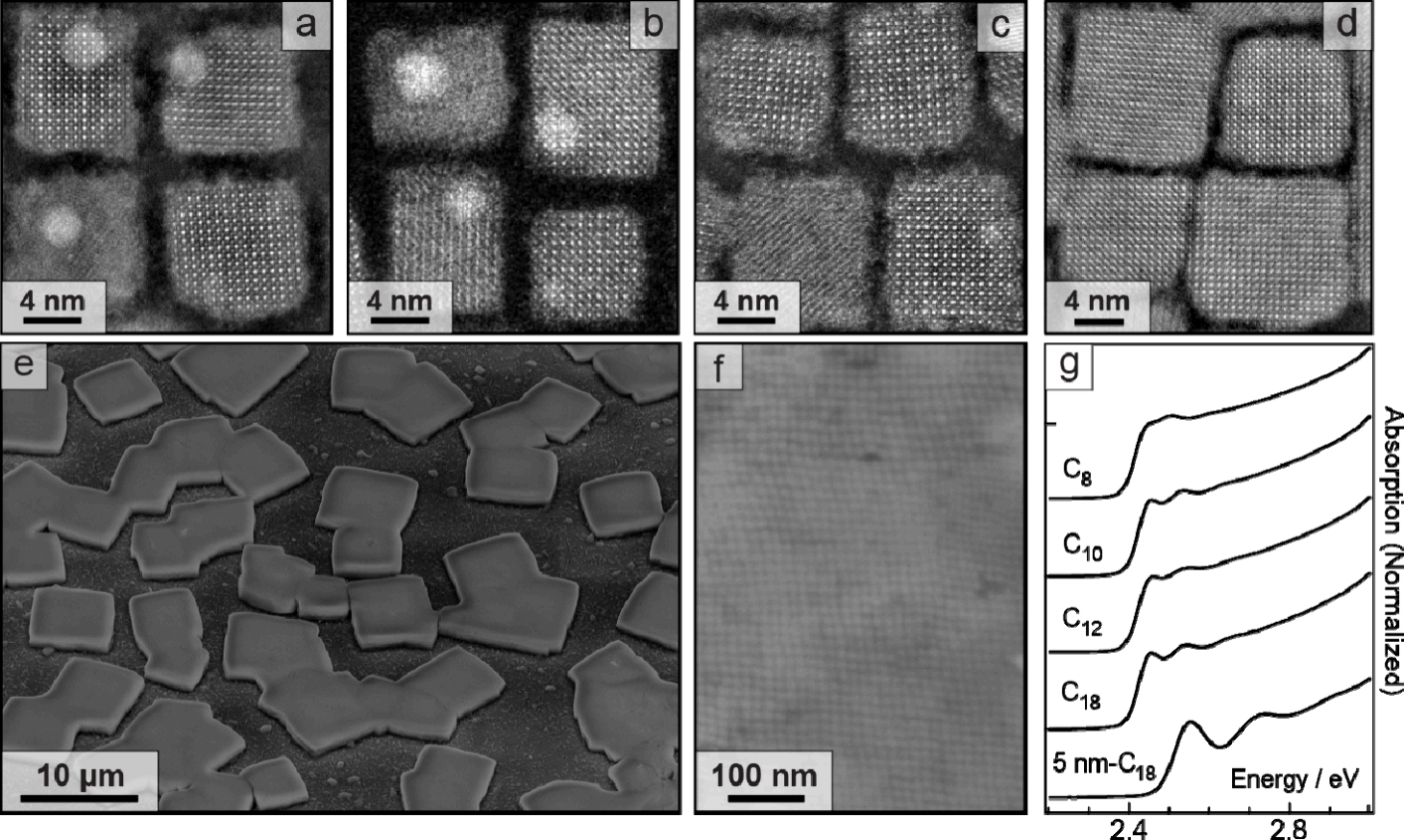
Nanocrystals with mixed ligands. (a–d) High-resolution STEM-HAADF images of CsPbBr_3_ nanocrystals capped with oleic acid and amines of different lengths. From left to right: (a) oleic acid - oleylamine (8 nm-C_18_), (b) oleic acid - dodecylamine (C_12_), (c) oleic acid - decylamine (C_10_), and (d) oleic acid - octylamine (C_8_). (e, f) Representative SEM images displaying a top view of 8 nm-C_18_ nanocrystal superlattices acquired with the substrate tilted 45° (e) and with no tilting (f). (g) Absorption spectra of toluene dispersions of nanocrystal samples.

**Table 1:** Summary of the GIWAXS Multilayer Diffraction Fits^a^

sample	*d* [Å]	*L* ± σ_L_ [Å]	*N* ± σ_**N**_ [planes]	*S* = *L*/(*d*·*N*)
Average between (001) and (100) Peaks				
5 nm-C_18_	5.884 ± 0.020	36.572 ± 2.044	8.890 ± 1.3030	0.70
8 nm-C_18_		37.708 ± 1.584	13.307 ± 1.660	0.48
C_12_		37.962 ± 1.306	12.627 ± 1.567	0.51
C_10_		33.786 ± 1.264	14.053 ± 1.449	0.41
C_8_		32.931 ± 1.182	18.759 ± 3.258	0.30
(101) peak				
C_10_	4.106 ± 0.003	26.477 ± 1.853	≈16	-
C_8_		29.994 ± 1.264	≈20	-

a Parameters: *d* = nanocrystal lattice constant; *L* = interparticle distance (surface to surface); σ_L_ = stacking disorder; *N* = nanocrystal thickness; and σ_N_ = nanocrystal thickness distribution. The average values are reported for brevity. For detailed fit parameters for each condition, see Tables S2, S3.

### GISAXS Evidence of Cumulative Disorder

The GISAXS and GIWAXS experiments described here were conducted at the ForMAX beamline^22^ of the MAX IV synchrotron. Experimental details and data processing are described in the Methods section. In our experiments, the beam height is equal to 50 μm, and the incident angles are 1.400° and 4.237°, with corresponding beam footprints of 2 mm and 0.68 mm, respectively. Since the typical edge length of superlattices ranges between 1 and 20 μm, all our measurements sampled many superlattices. Figure 2a–f displays the 2D GISAXS patterns and respective 1D slices of superlattice films grown from the dispersions of nanocrystals with mixed ligands shown in Figure 1. All GISAXS patterns exhibit well-defined spots indicative of the nanoscale 3D periodicity of the superlattices. The GISAXS patterns were indexed as cubic space group *Pm*3̅*m* using SUNBIM4.0 software,^23^ with a representative indexed pattern shown in Figure 2d. Figure 2f compares the in-plane (black curves) and out-of-plane (colored curves) 1D GISAXS profiles, from which the superlattice periodicities Λ = 2π/Δ*q* (Δ*q* = *q*_*n*+1_ – *q*_*n*_) were extracted (see Table S1). For 5 nm-C_18_, 8 nm-C_18_, C_12_ and C_10_ samples, identical periodicities in in-plane (*x*–*y* direction) and out-of-plane (*z*-direction) directions were obtained, consistent with nanocrystal sizes estimated from TEM and absorption spectra, once the ligand layer thickness is considered. For C_8_, a small difference between the in-plane and out-of-plane periodicities (139 vs 137 Å) can be rationalized by residual strain after solvent evaporation.

**Figure 2:**
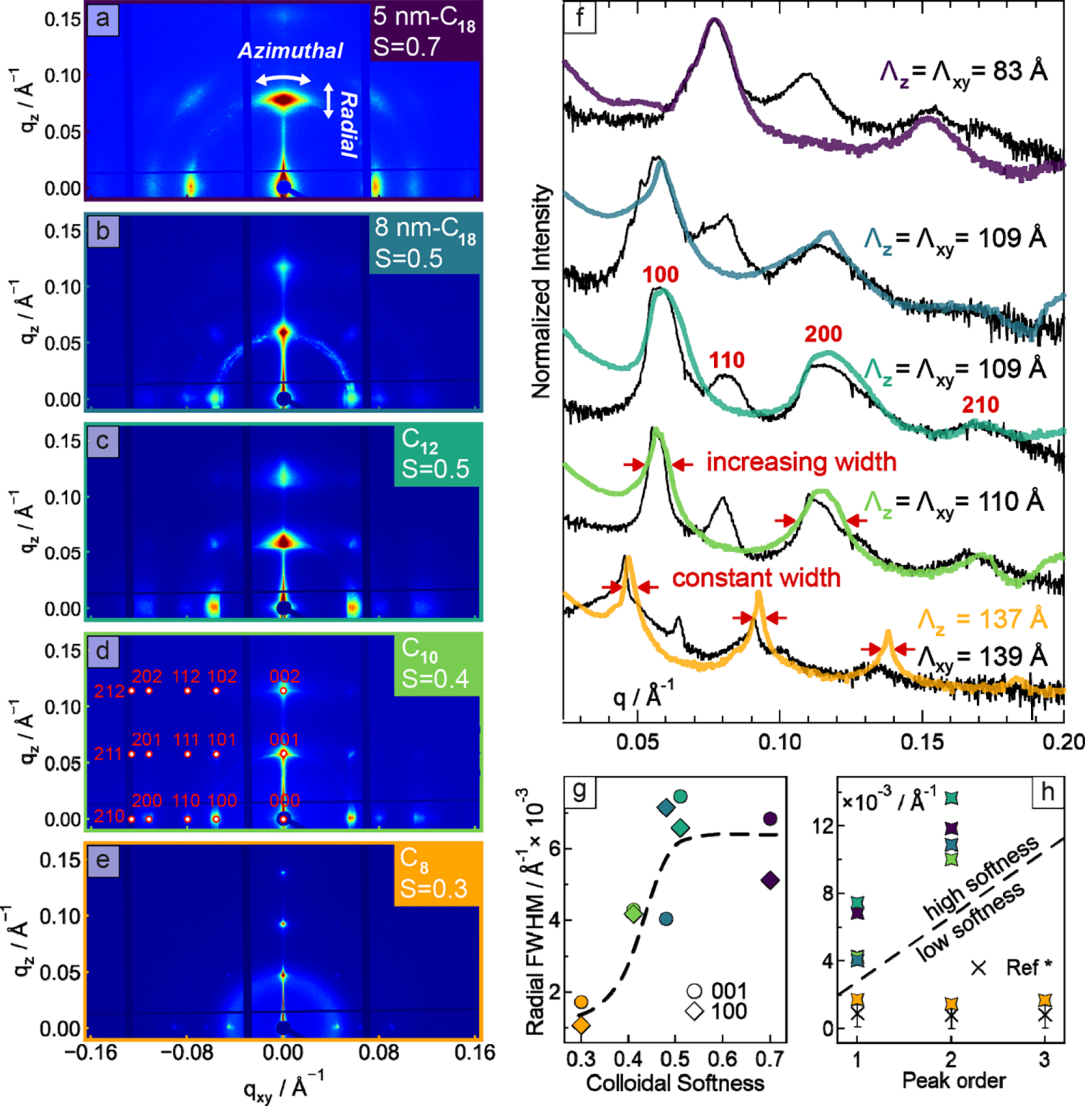
GISAXS characterization of superlattices from nanocrystals capped with mixed ligands. (a–e) Experimental GISAXS patterns from a series of nanocrystal superlattice samples, plotted on a logarithmic scale. From top to bottom: (a) 5 nm-C_18_ nanocrystals, (b) 8 nm-C_18_, (c) C_12_, (d) C_10_, and (e) C_8_. In (d), the red dots represent the simulated peaks from cubic indexing. Dark blue lines correspond to detector module gaps. (f) Intensity profiles extracted from the GISAXS patterns. Colored profiles are taken along out-of-plane “*z*” direction. Underlying black profiles are taken along the in-plane “*x*-*y*” direction. Each profile is an average of five slices centered in the origin (*q*_*xy*_ = *q*_*z*_ = 0). For each superlattice profile, the extracted periodicities Λ are reported (Λ = 2π/Δ*q*, where Δ*q* = *q*_*n*+1_ – *q*_*n*_). The arrows highlight the increasing vs constant peak width Δ*q*_*z*_ with diffraction order. (g) Radial broadening for the in-plane (001) and out-of-plane (100) directions. (h) Out-of-plane GISAXS peak broadening as a function of the peak order. The literature example of an amphiphile thin film with a lamellar phase is reported with “×” and extracted from the reference.^28^

The radial and azimuthal broadening of the first-order peaks (Figure 2g, see also Figures S3, S4, and S5) vary with the superlattice softness. Here, radial broadening carries information about nanoscale inhomogeneities in the superlattice periodicities and cumulative disorder, while azimuthal broadening indicates orientational disorder either at the level of whole superlattices or of multiparticle domains. Both radial and azimuthal broadening decrease from C_18_ to C_8_, as nanocrystals get bigger and ligands get shorter, indicating that a reduction in the nanocrystal softness increases the overall order of superlattices (see also Figure S6 and related discussion).

The GISAXS 2D patterns for all samples showed multiple spots, allowing us to compare how peak broadening changes with diffraction orders across different samples and use it as a qualitative distinction between cumulative and thermal-like nanocrystal displacements.^24–26^ We observed that the peak width increases with the diffraction order for all samples except C_8_ (as highlighted by the arrows in Figure 2f and plotted in Figure 2h). In this case, the resolution-limited width in GISAXS resembles crystalline multilayer systems (e.g., polyacene, amphiphilic crystals, and 2D Ruddlesden–Popper metal halides; see Figure 2h for comparison).^27–29^

### GIWAXS Evidence of Disorder Anisotropy

The as-synthesized CsPbBr_3_ nanocrystals used here as building blocks were established to have an orthorhombic structure (*Pbnm*, ICSD No. 98009-7851),^8^ and GIWAXS patterns were indexed using a pseudocubic notation for simplicity, as clarified in earlier works (Figure 3a shows a pattern with indexed spots relevant for the analysis).^8,9^ All GIWAXS patterns (Figure 3a–e) exhibit diffraction spots due to the fully oriented nanocrystals inside the superlattice, with the (100) and (001) spots showing characteristic satellites due to the structural coherence.^8–13^ To quantify cumulative disorder in multiple directions of the superlattice, we applied the multilayer diffraction routine developed for wide-angle θ:2θ X-ray diffraction to GIWAXS data. The main results are summarized in Table 1 and demonstrate shortening of the interparticle separation and increasing order with decreasing *S*. In addition to diffraction spots, the C_8_ sample pattern shows arcs coming from residual nonassembled nanocrystals, which were subtracted for analysis (Figures S7, S8, and S9 in the SI).

**Figure 3:**
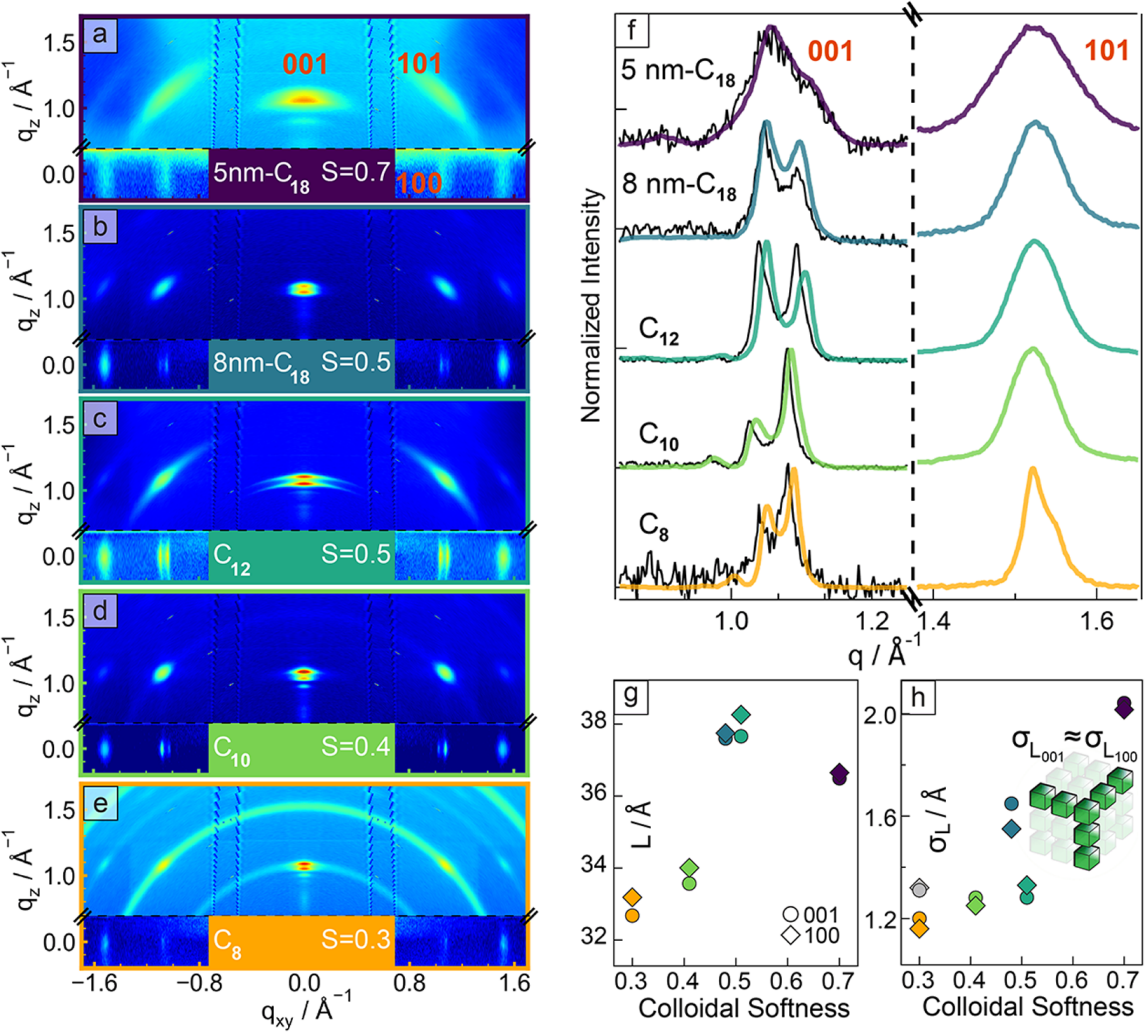
GIWAXS characterization of superlattices from nanocrystals capped with mixed ligands. (a–e) Experimental GIWAXS patterns from superlattice films of nanocrystals capped with mixed ligands. From top to bottom: (a) 5 nm-C_18_, (b) 8 nm-C_18_, (c) C_12_, (d) C_10_, and (e) C_8_. (f) Intensity profiles extracted from GIWAXS patterns. Colored traces represent the out-of-plane (001) and (101) peak profiles, with the underlying black profile representing the in-plane (100) one. (g, h) Internanocrystal distance (*L*) and average nanocrystal displacement (σ_L_), respectively. For the C_8_ sample (*S* = 0.3), gray points indicate fit values from the raw data before correction for the background of randomly oriented nanocrystals, and the orange points indicate fit values after correction. The cartoon inset in (h) depicts the isotropic order of nanocrystals along the three orthogonal directions.

Figure 3f displays normalized diffraction intensity profiles for the axial (100)/(001) and diagonal (101) peaks extracted from GIWAXS patterns. The multilayer diffraction fit parameters are summarized in Table S2, while the fits are shown in Figure S7. The interparticle spacing (*L*) and average nanocrystal displacement (σ_L_) decrease with the shortening of the amine ligand (Figure 3g,h, respectively), consistent with GISAXS results discussed above and prior characterization using a laboratory diffractometer.^14^ The progressive increase in superlattice order with decreasing softness can be rationalized by considering the forces involved in the self-assembly process. Typically, the effective pair potential established between the nanocrystals is a combination of attractive and repulsive contributions.^30^ The former includes the attraction between nanocrystal cores, which scales with the particle size, and the attraction between ligands, which increases with increasing ligand overlap and decreasing backbone separation. The repulsive potential instead includes osmotic and repulsive interactions, which have been predicted to scale with the length of the ligand.^31^ In addition, shorter ligands feature a steeper potential. Overall, both attractive and repulsive components reveal stronger and steeper effective pair potentials as a function of decreased softness. This could justify our experimental observation of shorter interparticle spacing and increased order with a decreased softness. In fact, larger particle size and shorter ligand length bring nanocrystals closer and reduce their displacement from the equilibrium position. A significant finding is that, for each sample, σ_L_ is comparable for the (001) peak in-plane and out-of-plane dimensions within error, indicating that the nanocrystal displacement parameters follow the cubic symmetry of the superlattice CsPbBr_3_ (Figure 3h). Slight differences in the atomic lattice constant and ligand interdigitation in the out-of-plane direction could be a further explanation of the difference in the two periodicities observed for the C_8_ superlattices in GISAXS.

### Diagonal Structural Coherence and the Need for a Different Model

The initial observations of structural coherence in perovskite superlattices were satellites of the (100) and (200) Bragg reflections of the pseudocubic perovskite structure, which align to the main lattice directions of the superstructure.^8,9^ Although (101) diffraction spots are visible in the GIWAXS patterns (Figure 3f), they completely lacked such intensity modulation satellites for the C_18_, C_12_, and C_10_ samples. This result contrasts with the 1D description of cumulative disorder implicit in the multilayer diffraction model because collective interference should monotonically fade at higher *q-*values, *q*(002) > *q*(101). Indeed, multilayer diffraction simulations performed for the (101) peak using the same σ_L_ value extracted for the (001) peak in Figure 3h indicate that collective interference modulations should be observed (see Figure S10). In contrast with this trend, the C_8_ sample showed a clear X-ray scattering intensity modulation of the (101) peak. Notably, in this case, the σ_L_ ≈ 1.26 Å value extracted from the (101) fit was compatible with the σ_L_ value observed for the main axial directions. For comparison, the C_10_ has lower σ_L_ in the axial direction (i.e., better structural coherence) but displays no interference modulation whatsoever for the (101) peak (σ_L_ > 2 Å, estimated limit of detection).

We hypothesized that the absence of such features for the (101) planes arises from their sensitivity to combinations of multiple displacement directions as opposed to (100) planes. This difference comes from geometric considerations: the (100) planes lie parallel to the nanocube facets, and their alignment with neighboring nanocrystals is determined by translation along a single Cartesian axis. In contrast, (101) planes are inclined 45° to the nanocube facets; therefore, their alignment is coupled to two translational degrees of freedom. As a result, displacement along one Cartesian axis disrupts only one of the three (100) families of planes but affects two of the three (101) families. Therefore, X-ray interference from the (101) planes of nanocubes is more easily suppressed by a slight positional displacement of nanocrystals in the superlattice. Although C_8_, C_10_, and C_12_ are all well-ordered superlattice samples from the (100) plane perspective, because of the clear presence of satellite peaks, high structural coherence in the {101} direction was detected only in C_8_. This can be rationalized by a loss in degrees of freedom (Figure 4c; see also Figure S11) with the increased rigidity of the nanocrystal packing as a consequence of low softness (*S* = 0.3 for C_8_, and 0.4 and 0.5 for C_10_ and C_12_, respectively). This interpretation ties with the higher peak sharpness observed in GISAXS for C_8_ superlattices compared to all other investigated samples, which also suggests that such assemblies are less prone to rotation and shear-like movement than softer superlattices, and therefore C_8_ is the most ordered superlattice either at the supracrystal scale (GISAXS) and at the subcrystal scale (GIWAXS).

**Figure 4:**
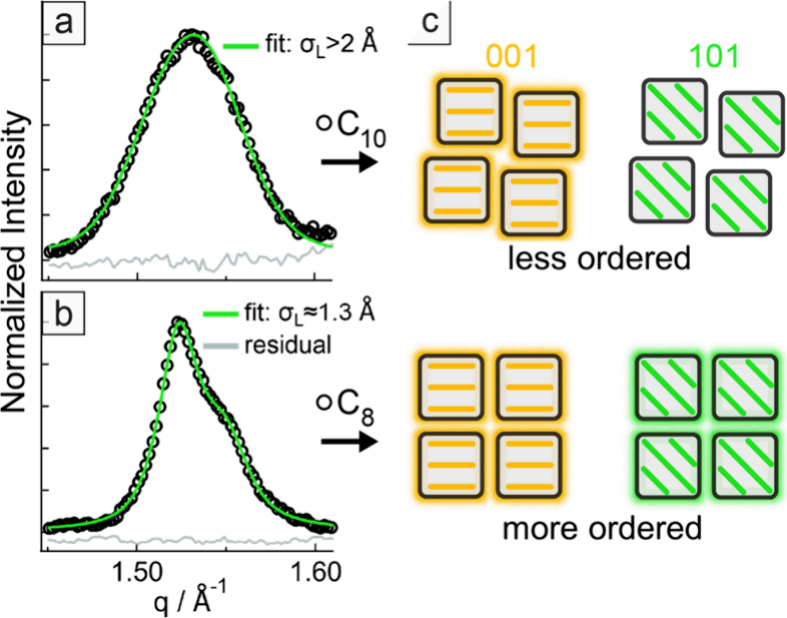
Experimental GIWAXS (101) peak profiles (circles, ca. *q*_center_ = 1.53 Å^–1^) and fits (continuous green lines) for (a) C_10_ and (b) C_8_ nanocrystal superlattice samples. The fit parameters are summarized in Table 1. (c) Illustration of the alignment (structural coherence) between (001) and (101) families of planes. In the case of more-ordered C_8_ nanocrystals, the alignment is maintained for the (001) and (101) families of planes, as highlighted by the glowing outlines. In the case of less-ordered C_10_ nanocrystals, the alignment is preserved for (001) but is lost for the (101) family of planes.

### A Sinusoidal Displacement Model

The experimental findings suggest that nanocrystals with the lowest colloidal softness produce superlattices, where structural coherence is better preserved in all directions of the lattice. That is consistent with prior observations of improved order in superlattices of large PbS nanocrystals^32^ and colloidal CsPbBr_3_ nanoplatelets with short octylamine ligands.^10^ The observation of multilayer interference modulating the (101) reflections in GIWAXS data was inconsistent with a simplified model of cumulative disorder where random displacements were added to the interparticle spacing at each nanocrystal site and were sufficient to reproduce multilayer interference from the family of (100) planes.^13,33^ In other words, if the first and second order (*h*00) Bragg reflections were modulated, then the (101) reflection should be modulated too, contradicting experimental observations.

To rationalize this discrepancy, a displacement model is needed that meets several key requirements. It must decouple the average face-to-face interparticle distance variability (captured in the multilayer diffraction model by the parameter σ_L_) from a shear-like lateral displacement. At the same time, the model should enforce correlation of various shifts across all of the spatial directions, preserve cumulative behavior, and prevent the nanocrystal position from diverging away from the superlattice origin. To meet these criteria, we introduced a periodic displacement (*u*) model, in which the nanocrystal coordinates are shifted by longitudinal (*u*_l_) and transversal (*u*_t_) sinusoidal displacements from the ideal positions (*u*_ideal_):

$$u=u_{\mathrm{ideal}}+u_{l}+u_{t}$$ 1

where

$$u_{\mathrm{ideal}}=\Lambda \left(i+j+k\right)$$ 2

$$u_{l}=A_{l}\left[\sin\left(\frac{2\pi \Lambda i}{\lambda_{l}}+\varphi \right)+\sin\left(\frac{2\pi \Lambda j}{\lambda_{l}}+\varphi \right)+\sin\left(\frac{2\pi \Lambda k}{\lambda_{l}}+\varphi \right)\right]$$ 3

$$u_{t}=A_{t}\left[\sin\left(\frac{2\pi \Lambda k}{\lambda_{t}}+\varphi \right)\;\cos\left(\frac{2\pi \Lambda j}{\lambda_{t}}+\varphi \right)+\sin\left(\frac{2\pi \Lambda i}{\lambda_{t}}+\varphi \right)\;\cos\left(\frac{2\pi \Lambda k}{\lambda_{t}}+\varphi \right)+\sin\left(\frac{2\pi \Lambda j}{\lambda_{t}}+\varphi \right)\;\cos\left(\frac{2\pi \Lambda k}{\lambda_{t}}+\varphi \right)\right]$$ 4

In the equations above, Λ is the superlattice periodicity, and *i*, *j*, *k* are the coordinates of the *n*th nanocrystal, *A*_l,t_ and λ_l,t_ are the amplitudes and wavelengths of the displacement, respectively, φ is a random phase shift. Longitudinal displacement alters the interparticle spacing along the edges and diagonals of the superlattice unit cell, leading to a similar broadening of collective interference features for both the (001)/(002) axial and (101) diagonal peaks. Conversely, the transversal displacement affects the displacement of nanocrystals in the plane perpendicular to the wave propagation vector, that is, parallel to the facet of neighboring nanocrystals. Figure 5c–e shows the wide-angle X-ray diffraction patterns calculated using the previously developed models^9,10,13^ with modifications for periodic displacements of nanocrystal coordinates as described in eqs 1–4, and either increasing *λ*_t_ from 400 to 600 nm or decreasing *A*_t_ from 3 to 5 nm. Both scenarios result in decreased disorder and lead to multilayer diffraction of the (101) reflection, while the other peaks remain unchanged. The same result would not have been achieved by tuning the parameters of the longitudinal displacement mode only (for example, by increasing *λ*_*l*_ from 200 to 300 nm or decreasing *A*_l_ from 0.3 to 0.2 nm; see Figure S12), which mostly affects the broadening of the (001) and (002) peaks (see also Figure S13). The main source of loss of structural coherence for the (101) reflection is the shear-like transversal oscillation, which shifts the nanocrystal perpendicular to the propagation of the displacement wave.

**Figure 5:**
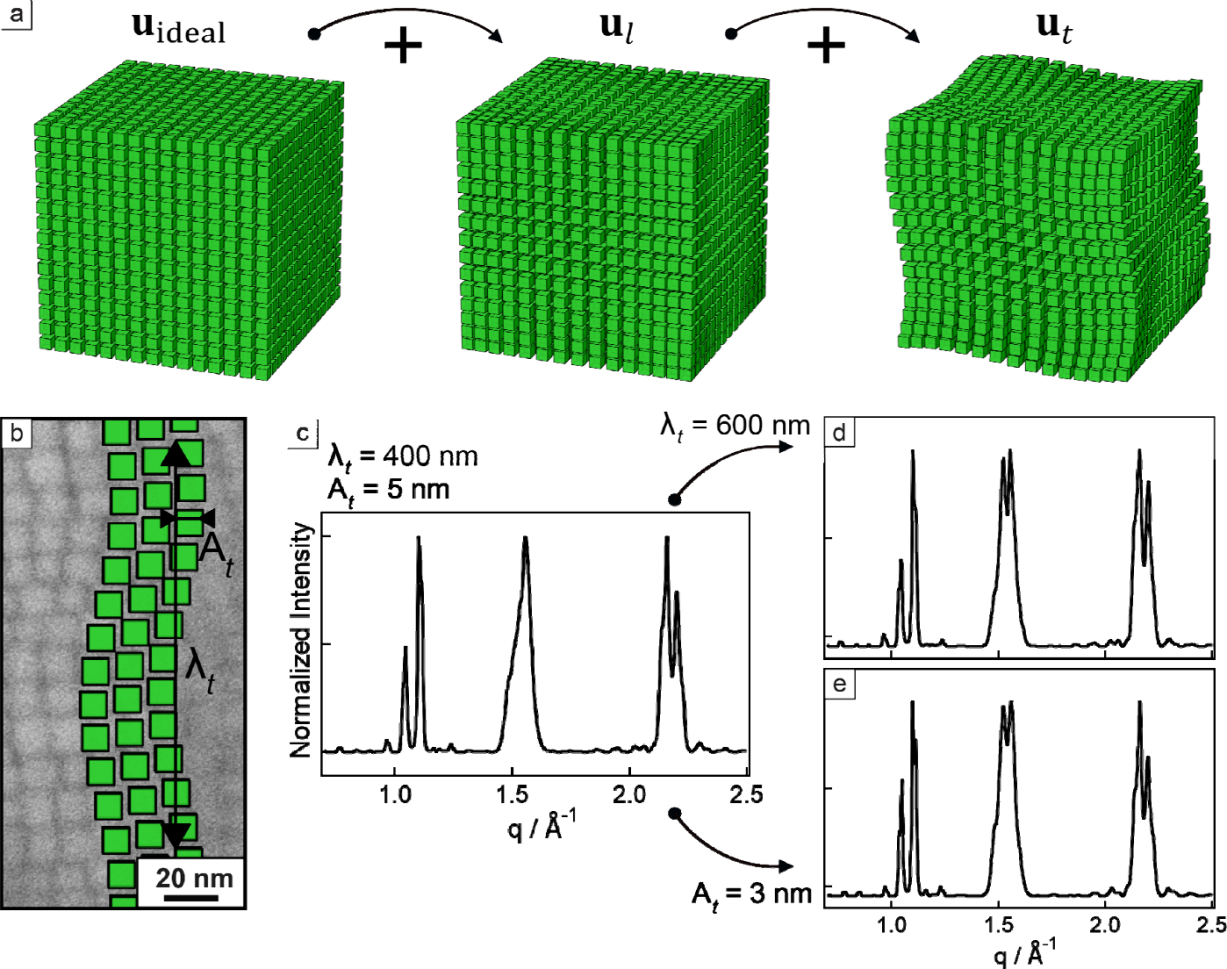
Periodic displacement in a nanocube superlattice. (a) Depicting the effect of the primary and secondary displacements on the packing of nanocrystals (eq 1). (b) High-resolution SEM image of a superlattice fragment showing wavy nanocrystal packing with an overlay illustrating its amplitude (*A*_t_) and wavelength (*λ*_t_). (c–e) Calculated wide-angle X-ray diffraction patterns of CsPbBr_3_ nanocrystal superlattices showing the effect of changing *λ*_t_ and *A*_t_ onto the multilayer interference of (101) peak.

We hypothesized that *A*_*t*_ is minimized in the C_8_ sample (*S* = 0.3) because large nanocrystals and short interparticle spacing restrict nanocrystal movement, dampening the amplitude of the periodic displacement. In contrast, softer nanocrystals (e.g., *S* = 0.4–0.7 for C_10_, C_12_, C_18_, and 5-C_18_ samples) exhibit more flexibility and thus erased {101} multilayer interference. Another interpretation is to view **u**_l_ and **u**_t_ as frozen waves that progressively displace nanocrystals from their equilibrium positions. In this analogy, **u**_l_ and **u**_t_ resemble static acoustic phonon modes with displacements parallel and perpendicular to the propagation direction, respectively. Superlattices can then be considered as a system of mass-springs with characteristic mass and spring constants *k*_l_ and *k*_t_ (longitudinal and transversal, respectively),^34^ where mass is that of a nanocrystal. The spring constants instead are connected to the ligand shape, length, and density, but their physical origins differ: *k*_l_ describes the response to deformation by compression and expansion, while *k*_t_ describes response to shear. Thus, considering Hooke’s law, reduced nanocrystal softness corresponds to a stiffer system with a longer spatial period of oscillation, analogous to the decrease in frequency of a harmonic oscillator with increasing mass.^35^ Experimentally, this is achieved with the C_8_ superlattice sample, which has the lowest softness and, thus, significantly suppressed shear. Wave-like displacements caused by various mechanisms can also be observed in other crystalline systems, such as martensitic phases,^36^ liquid crystals,^37,38^ and metal alloys.^39,40^ In liquid crystals, for example, twisted nematic or smectic structures, wave-like or helical, emerge due to molecular anisotropy, elasticity, or chirality, resulting in a periodic alignment of molecules.^41^ The wave-like nanocrystal packing in superlattices has been observed in SEM and TEM images in this work (see Figure 5b) as well as across the literature.^32,35,42–47^ It is therefore plausible that, similar to other ordered systems, ligand–ligand interactions and structural inhomogeneities in nanocrystal assemblies could give rise to periodic displacements.

## Conclusions

This study demonstrates that perovskite CsPbBr_3_ nanocrystals capped with mixed ligands can self-assemble into superlattices exhibiting high structural coherence in both the in-plane and out-of-plane directions. Through combining GISAXS and GIWAXS synchrotron measurements, we showed that the structural order in these superlattices systematically depends on colloidal softness, a tunable parameter defined by the ratio between ligand shell thickness and nanocrystal edge length. A counterintuitive observation of a broadening at wide angles (consistent with cumulative disorder) but resolution-limited peak broadening at small angles (suggesting an apparent thermal-like, uncorrelated disorder) in the nanocrystals with *S* = 0.3 prompted a deeper explanation. To rationalize these observations, we deconstructed nanocrystal displacements into longitudinal and transversal components, introducing sinusoidal modulation of nanocrystal coordinates that reconciles observed diffraction patterns and bridges regimes of thermal-like (where *A* → 0 and λ → ∞) and powder-like uncorrelated disorder (where *A* → ∞ and λ → 0). The model also offers a physical analogy to static acoustic phonon modes, linking decreased nanocrystal softness to a reduced displacement amplitude and longer spatial modulation periods. Such a wave-like deformation closely resembles the periodic structural modulations found in liquid crystals,^36–38,48^ reinforcing the analogy between nanocrystal superlattices and soft condensed matter systems. For example, a similar model has been used to describe the elasticity and disorder in granular solids.^49^ Although the sinusoidal displacement successfully reproduces the experimental findings, it might not be the only model that explains these phenomena. One of its strengths is that it ensures the same local disorder across the whole superlattice and accounts for different deformations – the requirements we considered essential in describing disorder in the nanocrystal superlattices. The periodic nanocrystal displacements invite compelling analogies with phonon modes and atomic vibrations and could be described by other models. As their exploration as quantum materials continues across nanoscience, this highlights the potential of nanocrystal superlattices as model systems for investigating excitonic and vibrational interactions^50–52^ and excitation transport.^4,53–55^ Findings of this work are an important step for understanding collective vibrations in CsPbBr_3_ nanoparticle assemblies.^34,56^ For example, it could lead to exploration of phonons and potential use of superlattices as acoustic metamaterials whose electronic properties and heat conductivity are tunable by nanocrystal softness and the resulting displacements in the superlattice.

## Methods

### Nanocrystal Synthesis and Characterization

Samples of 8–10 nm CsPbBr_3_ nanocrystals passivated with mixed ligands (8 nm-C_18_, C_12,_ C_10_, and C_8_ series) were synthesized by hot-injection following a previously reported procedure with minor variations.^14^ Briefly, the syntheses consisted of injecting 0.5 mL of a 0.073 M cesium oleate solution in 1-octadecene into the hot lead bromide (ca. 0.036 M PbBr_2_) solubilized in a mixture of alkylamine, oleic acid, and 1-octadecene. The temperature of the mixture was different for different amines (160 °C for 8 nm-C_18_, and 170 °C for C_8_, C_10_, and C_12_ amines). The as-synthesized nanocrystals were isolated either by centrifugation alone (C_18_ and C_12_) or with the aid of antisolvent ethyl acetate (C_10_ and C_8_). After isolation, the precipitate was redispersed in 300 μL of toluene, forming a concentrated dispersion, and centrifuged to remove undissolved material, and the obtained supernatant was used for subsequent characterization and superlattice growth. The quantum-confined nanocrystals of CsPbBr_3_ (5 nm-C_18_) were synthesized using the hot-injection synthesis of Dong et al.,^20^ with the detailed procedure reported by Gomes Ferreira et al.^21^ UV–vis optical absorption spectra were recorded on dilute dispersions of nanocrystals in toluene using Cary 500 (C_8_, C_10_, C_12_, and 8 nm-C_18_ samples) and PerkinElmer Lambda 1050 (5 nm-C_18_ sample) spectrometers. High-resolution scanning transmission electron microscopy (HRSTEM) images were acquired on a probe-corrected Thermo Fisher Spectra 30-300 STEM instrument operated at 300 kV. Images were acquired on a high-angle annular dark field (HAADF) detector with a current of 50 pA. SEM imaging was performed on superlattice films grown on top of silicon substrates using two instruments: a Zeiss GeminiSEM 560 (Zeiss, Oberkochen, Germany) field-emission gun operating at 10 kV acceleration voltage and a Helios G4 UX Dual Beam instrument operating at 10 kV.

### Synchrotron Experiments and Data Analysis

A thin film of nanocrystal superlattices for synchrotron experiments was prepared by drop-casting nanocrystal dispersions in toluene on silicon nitride substrates (1000 nm-thick SiN, Silson, product code M1000143, SiRN-5.0-200-2.5-1000) and leaving them to dry slowly, enclosed in a Petri dish. The nanocrystal concentration in the drop-cast dispersion was approximately 0.5 μM, to allow the growth of isolated superlattices. The concentrated nanocrystal dispersions obtained as described above are stable over the course of 7–10 days needed for shipping and handling at the synchrotron and do not show visible signs of degradation during handling under ambient conditions. GISAXS and GIWAXS experiments were performed using the ForMAX beamline at the MAX IV Laboratory.^22^ For GISAXS, ForMAX employs an Eiger2 4M detector in an 8 m vacuum vessel, while GIWAXS uses a custom windmill-shaped detector with a central aperture for GISAXS transmission. In the GISAXS/GIWAXS experiments, incident angles (α_*i*_) of 1.400° and 4.237° were used with a 14.31 keV beam, providing approximately 5.5 × 10^14^ photons/s total flux. The 4.237° incidence angle was employed to collect the 2D map from which the 101 peak profiles were extracted and reported in Figure 4 for the C_10_ and C_8_ superlattices and in Figure S11 for all other superlattices. The beam size was 50 × 50 μm^2^ at normal incidence (with beam height *H* equal to 50 μm), which corresponds to the elongated footprint *L*_f_ of the beam on the sample with a length of ca. 2 mm for 1.400° incident angle and ca. 680 μm for 4.237° incident angle, estimated considering the relationship *L*_f_ = *H/*sin­(*α*_*i*_). The sample-to-detector distances were 2990 mm for GISAXS and 142 mm for GIWAXS. Data calibration for GISAXS and GIWAXS was performed by employing silver behenate and LaB_6_ powders as the standards, respectively. The GISAXS patterns were indexed using SUNBIM4.0 software.^23^ The 1D profiles were extracted from the 2D maps by averaging 5 pixel lines in correspondence with the desired spots.

## Supplementary Material



## Notes

Additional electron microscopy characterization, summaries of multilayer diffraction fits, GISAXS and GIWAXS analyses, calculated diffraction patterns (PDF)
