# Supplementary material for: Sinusoidal Displacement Describes Disorder in CsPbBr3 Nanocrystal Superlattices
Source: ACS Nano. 2026 Jan 17;20(4):3867–77. doi: 10.1021/acsnano.5c20745 (PMC12874646; doi:10.1021/acsnano.5c20745)
Supplement: Supplementary file 1 [file nn5c20745_si_001.pdf]

Supporting Information for

Sinusoidal Displacement Describes Disorder in $CsPbBr_3$ Nanocrystal Superlattices

Umberto Filippi[1,2]*, Stefano Toso[3,4]*, Matheus G. Ferreira[3], Lorenzo Tallarini[3], Yurii P. Ivanov[1], Francesco Scattarella[5], Simone Lauciello[1], Vahid Haghighat[6], Huaiyu Chen[7], Megan O. Hill Landberg[6], Giorgio Divitini[1], Jesper Wallentin[7], Cinzia Giannini[5]*, Liberato Manna[1]*, Dmitry Baranov[3]*

[1]Istituto Italiano di Tecnologia, Via Morego 30, 16163 Genova, Italy
[2]International Doctoral Program in Science, Università Cattolica del Sacro Cuore, Brescia 25121, Italy
[3]Division of Chemical Physics and NanoLund, Department of Chemistry, Lund University, P.O. Box 124, SE-221 00 Lund, Sweden
[4]Department of Chemical Engineering, Massachusetts Institute of Technology, Cambridge, Massachusetts 02139, United States
[5]Istituto di Cristallografia (CNR-IC), via Amendola 122/O, Bari 71026, Italy
[6]MAX IV Laboratory, Lund University, 22100 Lund, Sweden
[7]Synchrotron Radiation Research and NanoLund, Department of Physics, Lund University, 22100 Lund, Sweden

E-mail: umberto.filippi@iit.it, stefano.toso@chemphys.lu.se, cinzia.giannini@cnr.it, liberato.manna@iit.it, dmitry.baranov@chemphys.lu.se

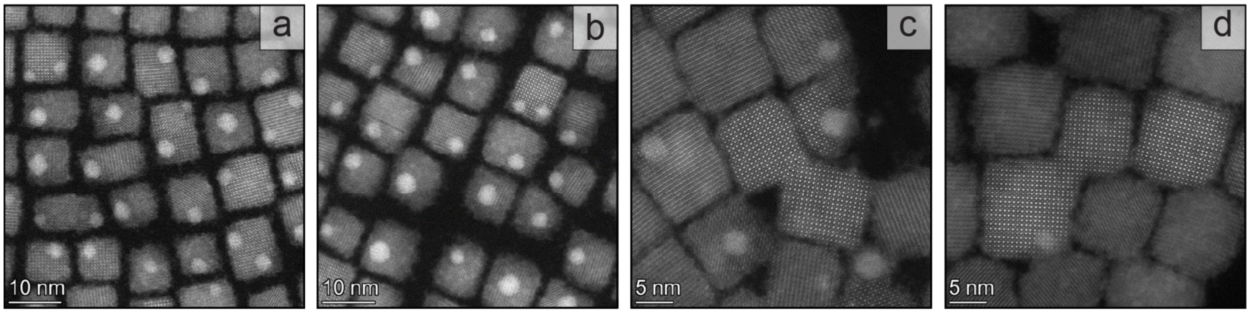

Figure S1. High-resolution STEM-HAADF (High-Angle Annular Dark Field) images of monolayers of (a),(b) 8nm-C_{18} nanocrystals (well-separated from each other) and (c),(d) C_8 nanocrystals (some necking occurs under the imaging conditions).

Table S1. Superlattice periodicities from the GISAXS profiles (Figure 2f in the main text).

	**Periodicity [Å]**		**Crystal System**	**Space group**
**Sample**	**Out-of-plane**	**In-plane**		
**C_{18} 5 nm**	82.8 ± 0.8		Cubic	Pm-3m
**C_{18} 8 nm**	109.1±1.3		Cubic	Pm-3m
**C_{12}**	108.6±1.2		Cubic	Pm-3m
**C_{10}**	109.9±1.2		Cubic	Pm-3m
**C_8**	136.8 ± 0.3	139.1 ± 0.3	Cubic	Pm-3m

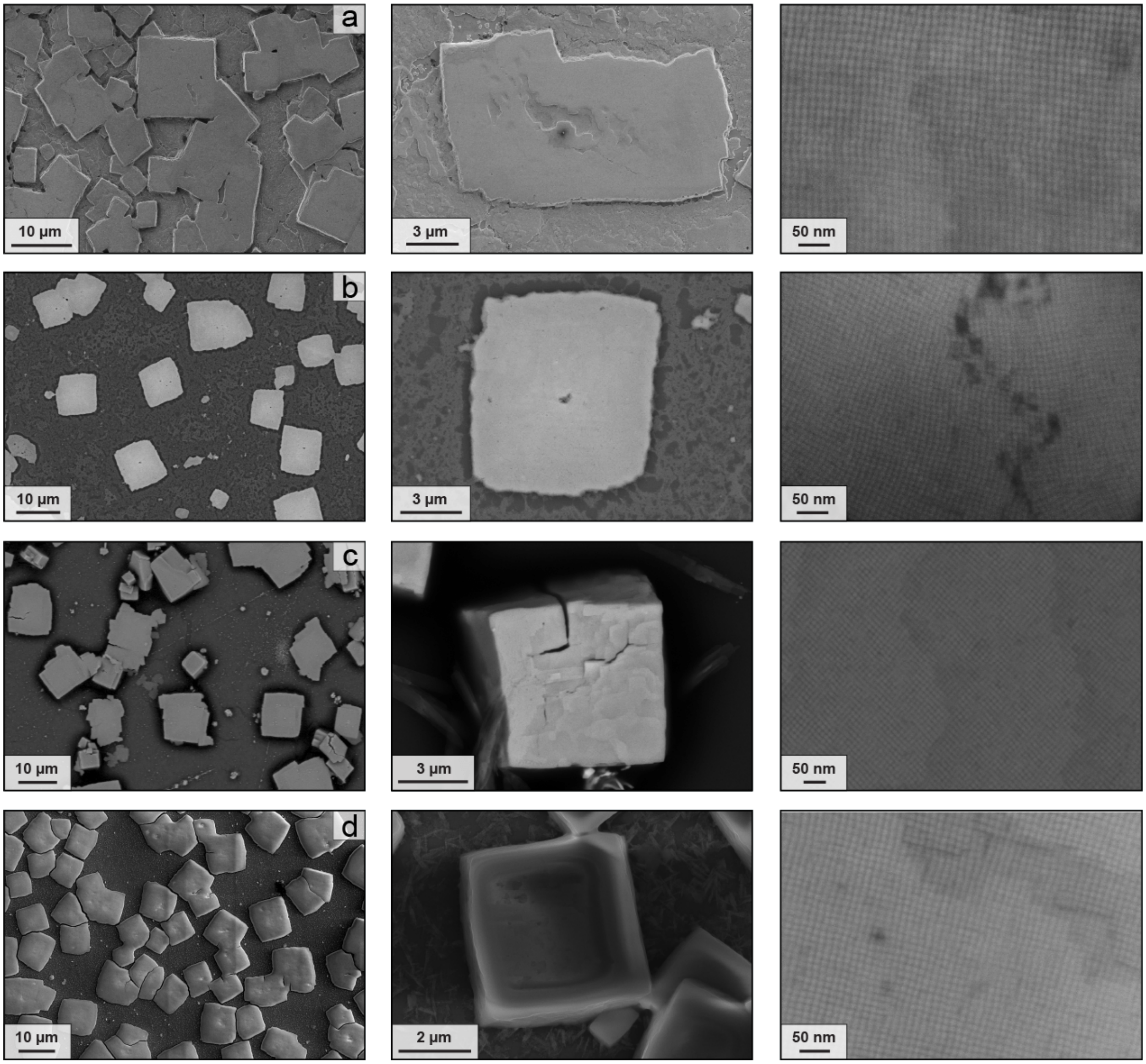

Figure S2. High-resolution SEM images of superlattices assembled from (a) C_8-capped nanocrystals, (b) C_{10}-capped nanocrystals, (c) C_{12}-capped nanocrystals and (d) 8nm-C_{18} nanocrystals.

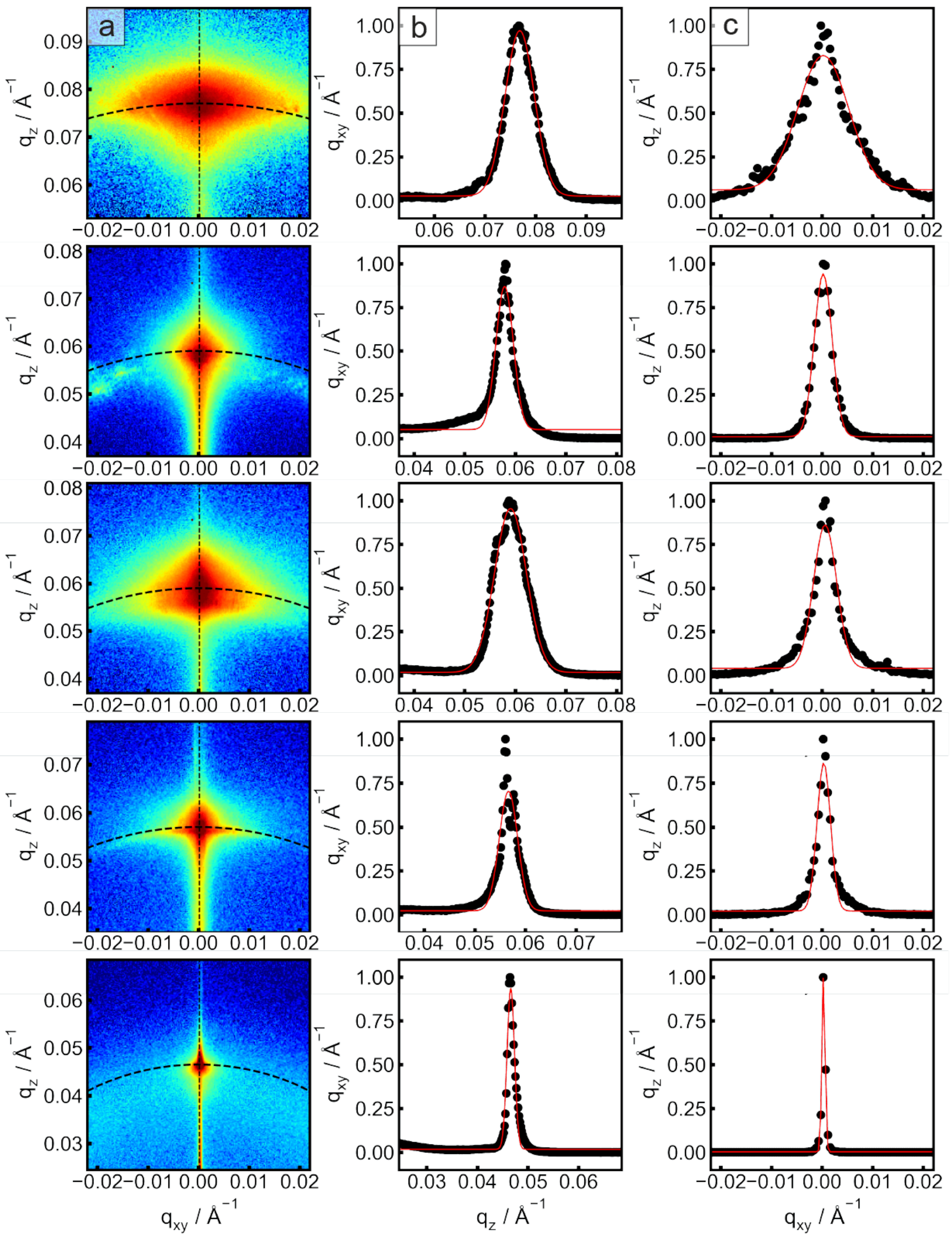

Figure S3. (a) 001 GISAXS peak, (b) azimuthal broadening profile and Gaussian fitting, (c) radial broadening profile and Gaussian fitting. From top to bottom: 5nm-C_{18}, 8nm-C_{18}, C_{12}, C_{10}, and C_8 nanocrystal superlattice samples.

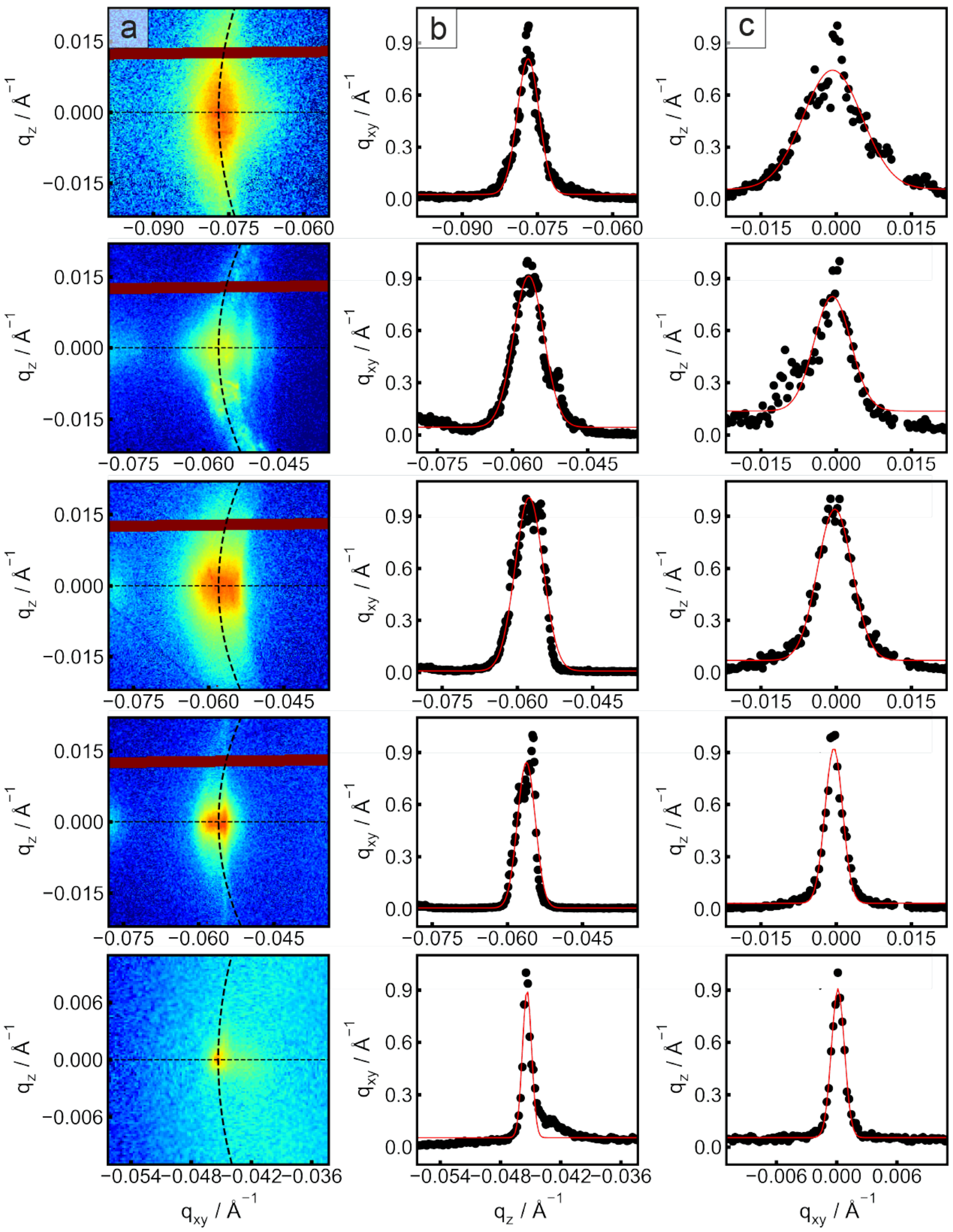

Figure S4. (a) 100 GISAXS peak, (b) azimuthal broadening profile and Gaussian fitting, (c) radial broadening profile and Gaussian fitting. From top to bottom: 5nm-C_{18}, 8nm-C_{18}, C_{12}, C_{10}, and C_8 nanocrystal superlattice samples.

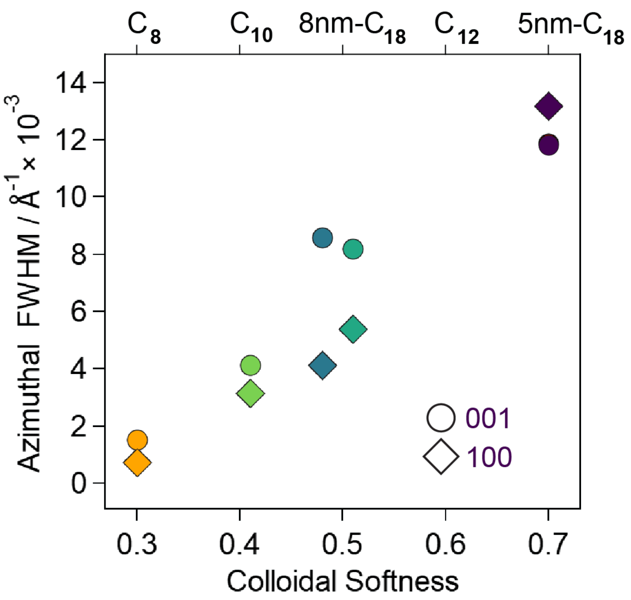

Figure S5. GISAXS azimuthal broadening for in-plane (100) and out-of-plane (001) directions.

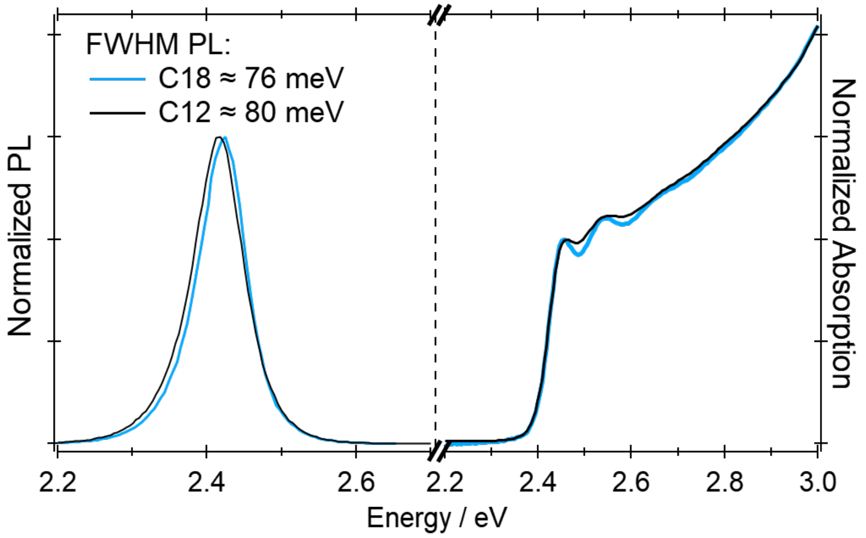

Figure S6. Comparison of the photoluminescence (PL) and absorption spectrum of C_{12} and C_{18} nanocrystals dispersed in toluene. The wider PL spectrum and less pronounced excitonic absorption peaks indicate a larger size dispersion for C_{12} nanocrystals than for 8nm-C_{18} nanocrystals. We hypothesize that the larger size dispersion of the C_{12} sample leads to pronounced size selectivity and the presence of superlattices with different periodicities. This could further explain the large value of the radial broadening of the GISAXS peaks reported in Figure 2 of the main text for the C_{12} sample. The enhanced size selectivity in nanocrystal superlattices with shorter ligands was reported previously.[1]

Table S2. Multilayer diffraction fit results of GIWAXS patterns. Structure parameters extracted from the multilayer diffraction fits. The fits are shown in Figure S7. To account for the disordered contribution present in the C_8 GIWAXS pattern (Figure 3e), we subtracted a Gaussian background from the peak profile; the fitting results before and after subtraction are reported in *C8 and C8 rows, respectively. Parameters: d = nanocrystal lattice constant; L = interparticle distance (surface to surface); σ_L = stacking disorder; N = nanocrystal thickness; σ_N = nanocrystal thickness distribution, S – colloidal softness [calculated as L/(d·N)].

Sample	d [Å]	L [Å]	σ_L [Å]	N [planes]	σ_N [planes]	S = L/(d·N)
**(001) Out-of-plane**						
**5nm-C_{18}**	5.886	36.493	2.041	8.879	1.239	0.70
**8nm-C_{18}**	5.866	37.814	1.567	13.531	1.561	0.48
**C_{12}**	5.852	37.661	1.282	12.249	2.129	0.53
**C_{10}**	5.866	33.567	1.281	13.938	2.012	0.41
**C_8**	5.860	32.677	1.203	19.116	3.501	0.29
***C_8**	5.858	32.677	1.325	19.212	2.872	0.29
**(100) In-plane**						
**5nm-C_{18}**	5.906	36.650	2.046	8.901	1.367	0.70
**8nm-C_{18}**	5.910	37.601	1.601	13.082	1.759	0.49
**C_{12}**	5.898	38.262	1.330	13.004	1.004	0.50
**C_{10}**	5.891	34.005	1.246	14.167	0.886	0.41
**C_8**	5.905	33.185	1.160	18.401	3.014	0.31
***C_8**	5.909	33.126	1.31	18.343	3.504	0.31

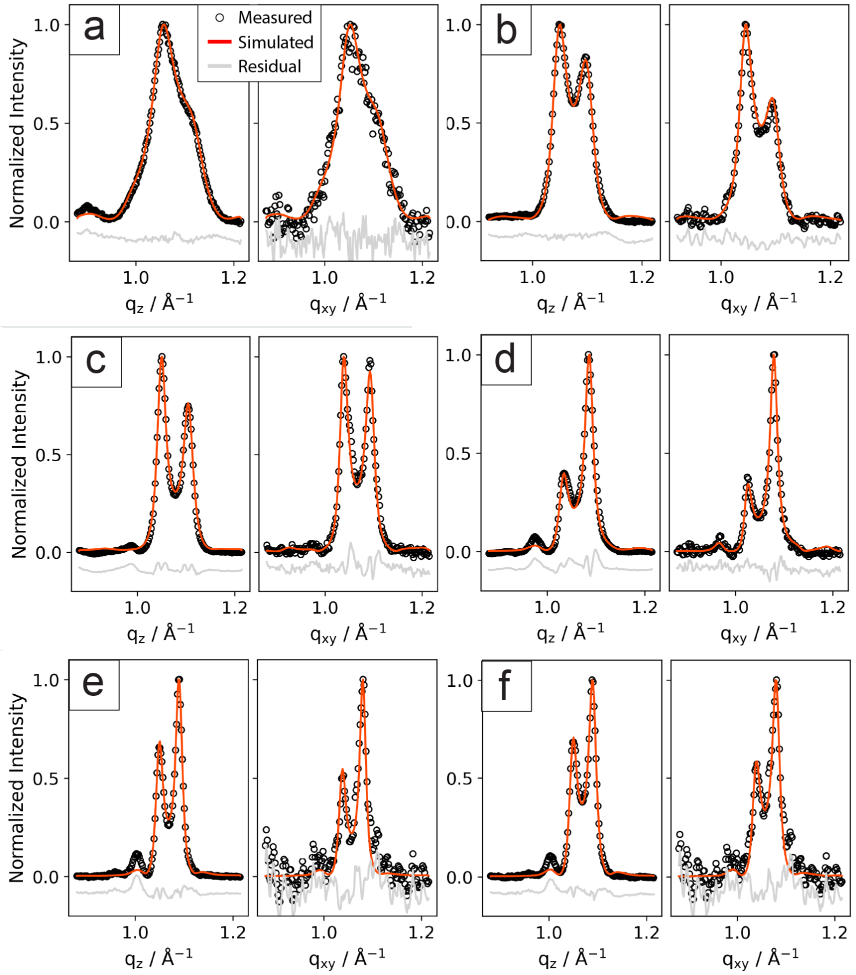

Figure S7. Multilayer diffraction fits of GIWAXS patterns. a-f) Out-of-plane (001) (left) and in-plane (100) (right) diffraction profiles extracted from the 2D GIWAXS patterns reported in Figure 3. They correspond to (a) 5nm-C_{18}, (b) 8nm-C_{18}, (c) C_{12}, (d) C_{10} and (e) C_8, and (f) *C_8.

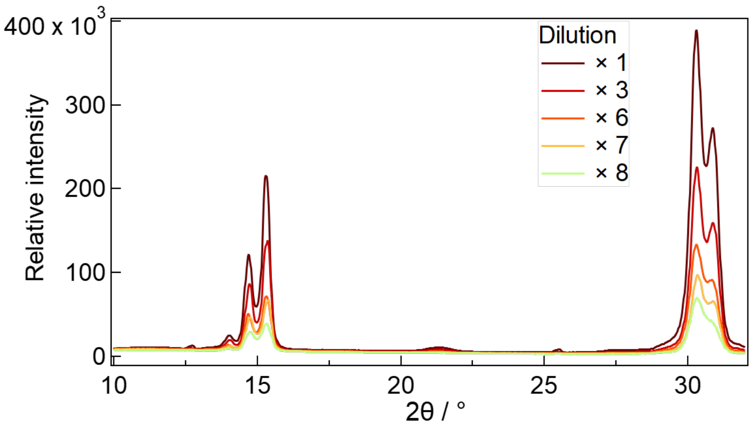

Figure S8. Concentration-dependent C_8 superlattice order.

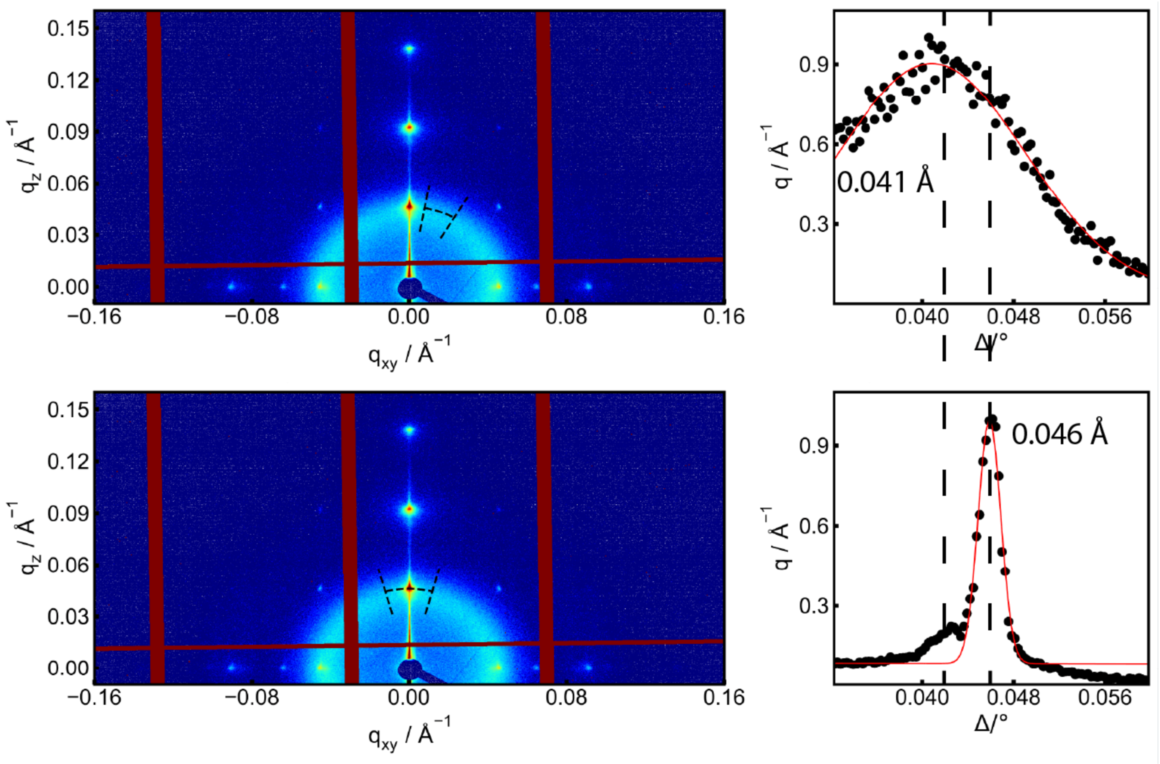

Figure S9. GISAXS C_8 fitting of the disordered component.

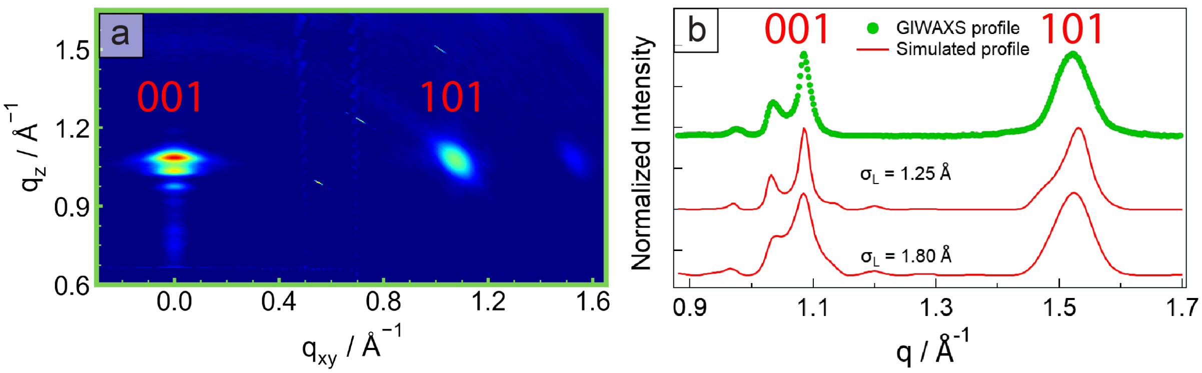

Figure S10. (a) 2D GIWAXS pattern of C_{10} superlattice and (b) comparison between the 001 and 101 profiles extracted from (a) and the calculated diffraction patterns of C_{10} superlattice with $\sigma_L \approx 1.28$ and 1.8 Å.

Table S3. Multilayer diffraction fit results of Bragg patterns. Structure parameters extracted from the multilayer diffraction fits. Fits are shown in Figure S11.

**Sample**	**d [Å]**	**L [Å]**	**σ_L [Å]**	**N [planes]**	**σ_N [planes]**
**(001) Out-of-plane**					
**5 nm-C_{18} NCs**	5.879	36.483	2.040	8.831	1.366
**8 nm-C_{18} NCs**	5.828	36.359	1.510	12.743	1.998
**C_{12}**	5.835	36.912	0.866	12.762	0.746
**C_{10}**	5.808	32.905	0.958	13.778	0.573
**C_8**	5.808	32.901	1.203	19.116	3.501
**(101) Out-of-plane**					
**C_{10}**	4.108	26.477	1.853	≈16	n/a
**C_8**	4.103	29.994	1.264	≈20	n/a

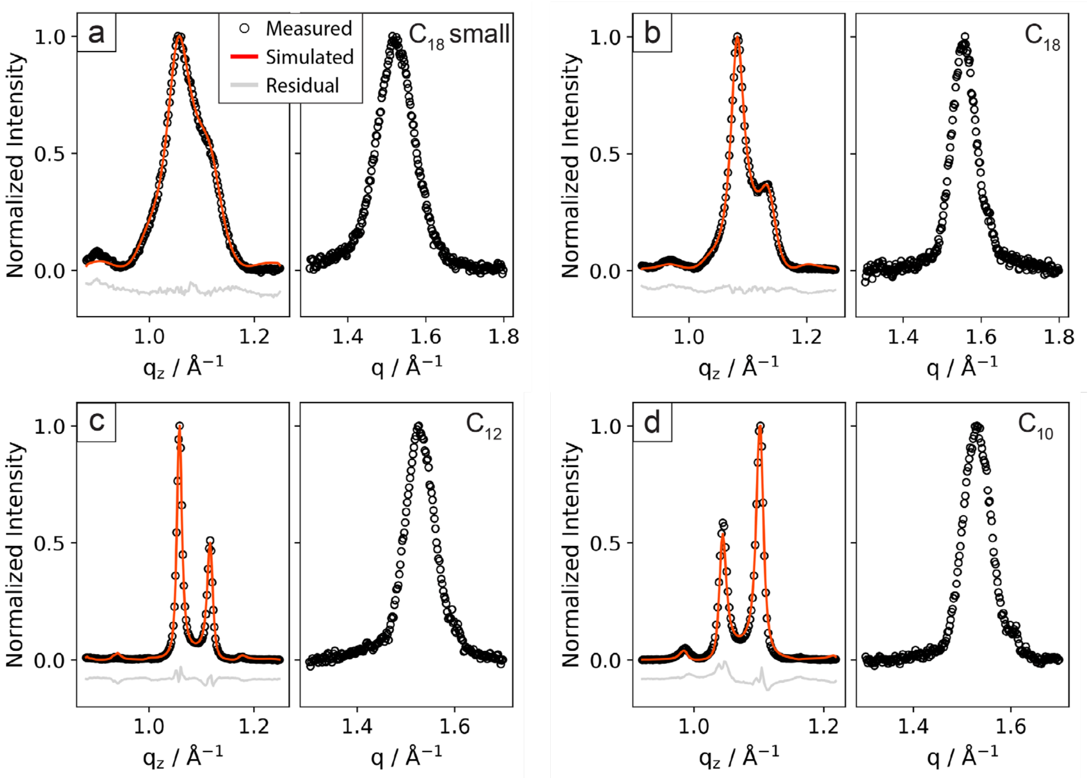

Figure S11. Multilayer diffraction fits of Bragg patterns. a-d) On the right are (001) out-of-plane peak profiles and on the left are (101) peak profiles extracted from the 2D GIWAXS patterns.

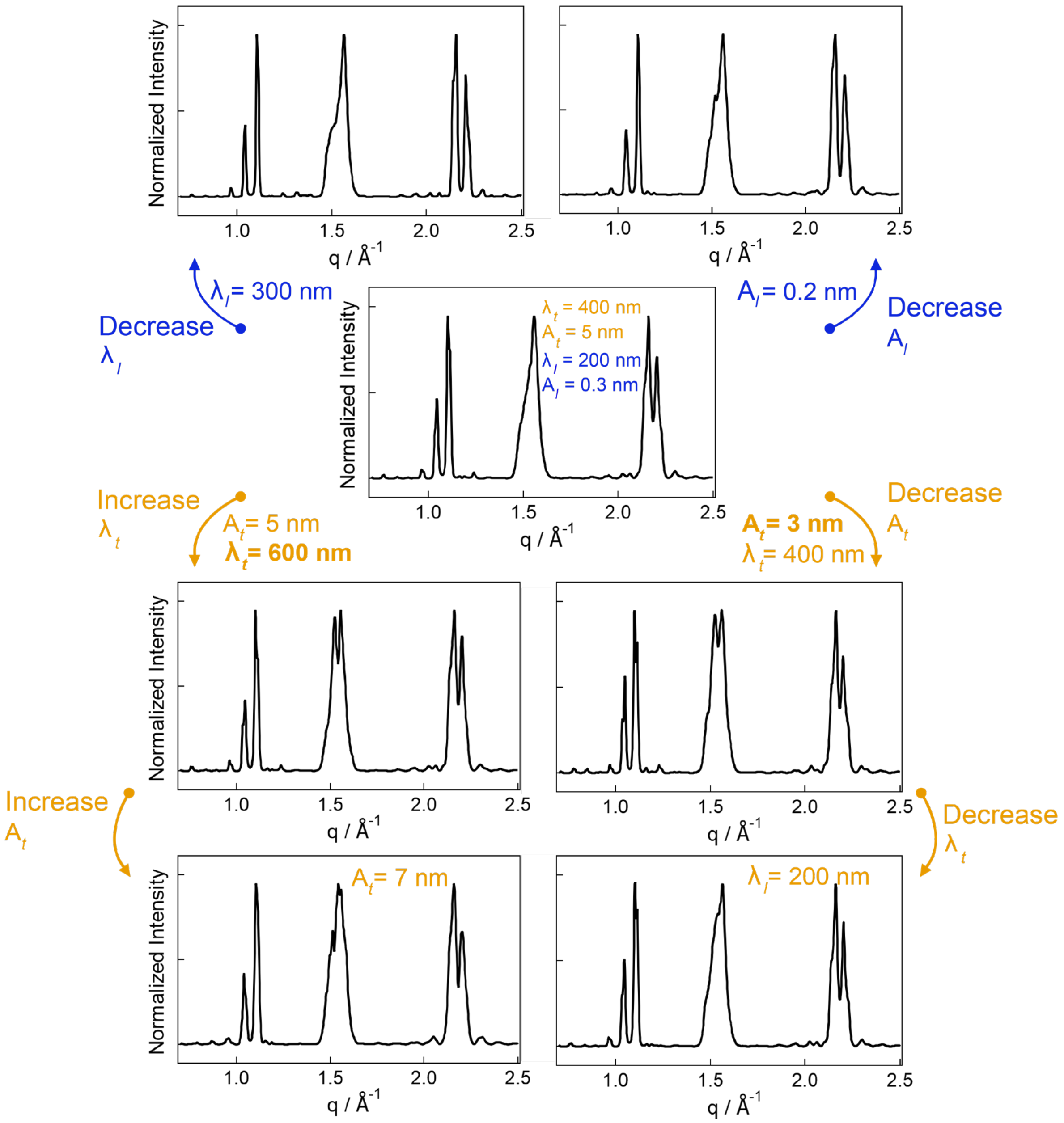

Figure S12. Simulated wide-angle X-ray diffraction patterns of $CsPbBr_3$ nanocrystal superlattices showing the effect of changing λ_l (longitudinal wavelength), A_l (longitudinal amplitude), λ_t (transversal wavelength) and A_t (transversal amplitude). The longitudinal parameter has little effect on the (101) peak, while it affects the broadening of the (001) and (002) peaks. As discussed in the main text, the broadening of the (101) peak is primarily influenced by the transversal parameters.

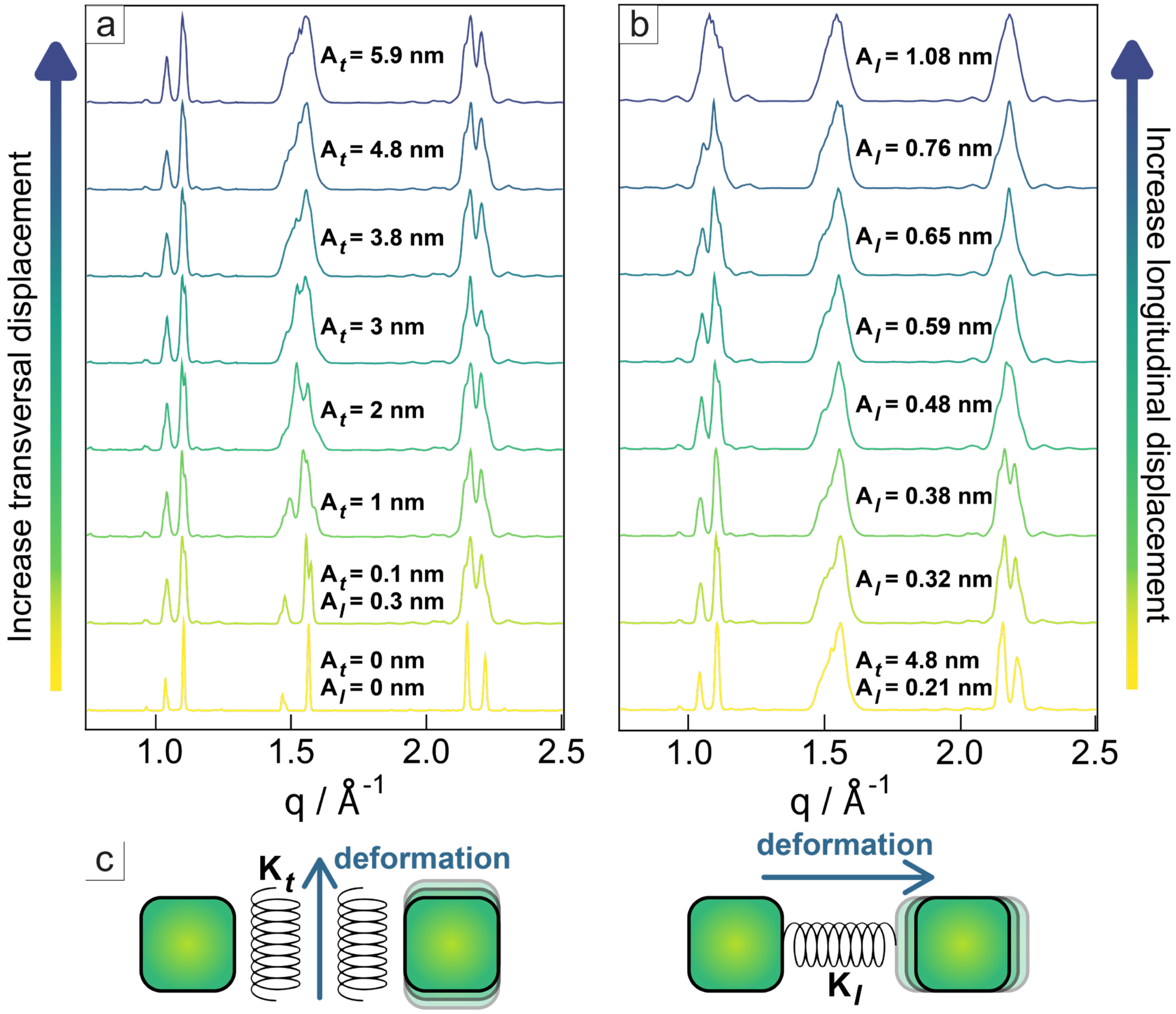

Figure S13. (a, b) Simulated wide-angle X-ray diffraction patterns of $CsPbBr_3$ nanocrystal superlattices showing the effect of changing A_t (transversal amplitude) and A_l (longitudinal amplitude) respectively (keeping constant the oscillation wavelengths $\lambda_t =$ 400 nm and $\lambda_l =$ 200 nm). These simulations explore the extreme regimes characterized by oscillations with zero amplitude (bottom pattern in (a)) or extremely large amplitudes (top patterns in (a) and (b)). (c) Illustration of the transversal (left) and longitudinal (right) spring constants, k_t and k_l. k_l describes the compressibility of ligands when nanocrystals are pushed towards each other, and k_t describes the shear when nanocrystals are sliding parallel to each other.
